# NFATC2 Modulates Radiation Sensitivity in Dermal Fibroblasts From Patients With Severe Side Effects of Radiotherapy

**DOI:** 10.3389/fonc.2020.589168

**Published:** 2020-12-16

**Authors:** Joshua Dulong, Clara Kouakou, Yasmina Mesloub, Julie Rorteau, Sandra Moratille, Fabien P. Chevalier, Tatiana Vinasco-Sandoval, Michèle T. Martin, Jérôme Lamartine

**Affiliations:** ^1^ Laboratory of Tissue Biology and Therapeutic Engineering, CNRS UMR5305, University of Lyon, Claude Bernard University Lyon I, IBCP, Lyon, France; ^2^ CEA, Genomics and Radiobiology of Keratinopoiesis, DRF/IBFJ/iRCM, Université Paris-Saclay, Evry, France

**Keywords:** radiotherapy, radiosensitivity, human skin fibroblasts, transcriptome, NFATC2, normal tissue side effects

## Abstract

Although it is well established that 5 to 15% of radiotherapy patients exhibit severe side-effects in non-cancerous tissues, the molecular mechanisms involved are still poorly known, and the links between cellular and tissue radiosensitivity are still debated. We here studied fibroblasts from non-irradiated skin of patients with severe sequelae of radiotherapy, to determine whether specific basal cell activities might be involved in susceptibility to side-effects in normal tissues. Compared to control cells, patient fibroblasts exhibited higher radiosensitivity together with defects in DNA repair. Transcriptome profiling of dermal fibroblasts from 16 radiotherapy patients with severe side-effects and 8 healthy individuals identified 540 genes specifically deregulated in the patients. Nuclear factor of activated T cells 2 (*NFATC2*) was the most differentially expressed gene, poorly expressed at both transcript and protein level, whereas the *NFATC2* gene region was hypermethylated. Furthermore, *NFATC2* expression correlated with cell survival after irradiation. Finally, silencing *NFATC2* in normal cells by RNA interference led to increased cellular radiosensitivity and defects in DNA repair. This study demonstrates that patients with clinical hypersensitivity also exhibit intrinsic cellular radiosensitivity in their normal skin cells. It further reveals a new role for NFATC2 as a potential regulator of cellular sensitivity to ionizing radiation.

## Introduction

Radiosensitivity is the relative sensitivity of cells, tissues, organs, and organisms to the injurious effects of ionizing radiations. This notion includes very different outcomes according to the scale at which it is analyzed. At cellular level, sensitivity to ionizing radiation is notably evaluated by the rate of immediate or delayed death in irradiated cell cultures and by cell capacity to repair DNA damage. At organism level, radiosensitive individuals are those that develop severe effects in irradiated tissue whereas the majority of the population exposed to the same dose show no or only mild effects. This is particularly obvious in the context of radiotherapy (RT), the main source of human exposure to high-dose ionizing radiation, where 5–15% of patients exhibit severe side-effects in irradiated normal tissues (1, 2), including fibrosis (3), necrosis, and sometimes radio-induced secondary cancers (4, 5). A central question regarding the multi-scale nature of radiosensitivity is whether intrinsic cellular radiosensitivity is a mirror of organism sensitivity. If that is the case, cellular testing would be a means of identifying radiosensitive patients and predicting deleterious outcome of radiation exposure. Moreover, using cultured cells as models of radiation sensitivity would shed light on the intrinsic mechanisms, which are far from clear. However, to date, data on the correlation between cellular and organism radiosensitivity are contradictory, especially regarding skin fibroblasts, a cell type which has been directly involved in the development of radiotherapy side-effects (6). Previous studies reported higher radiation toxicity in dermal fibroblasts from radiosensitive patients (7–11) and a correlation between clinical grades and cellular radiosensitivity (12, 13). However, other authors reported no difference in dermal fibroblast radiosensitivity between radiosensitive and radio-tolerant individuals (14, 15).

To further investigate this question, we used cutaneous fibroblasts from a collection of RT patients exhibiting severe side-effects of radiotherapy, that were classified according to side-effect severity (16). The first goal of this study was to investigate the link between cellular and individual radiosensitivity. The second goal was to shed light on the complex molecular mechanisms of the cellular response to ionizing radiation.

Here, we investigated the radiation toxicity and the DNA repair ability of skin fibroblasts from patients with RT severe side effect and we observed that patient fibroblasts exhibited higher cell death and profound DNA repair defects compared to normal control cell samples. By a transcriptomic analysis, we identified the transcription factor NFATC2 as being strongly repressed in patient fibroblasts, with hypermethylation on the coding sequence. Finally, we demonstrate that the repression of NFATC2 is able to increase the radiation sensitivity of normal fibroblasts, suggesting that this protein is involved in the establishment of the radiosensitive phenotype.

## Materials and Methods

### Cell Culture

Fibroblasts from patients were obtained from the INSERM UMR1052 COPERNIC cell collection (16). This collection was approved by the regional Ethical Committee (CPP Sud-Est, Lyon, France) and cell lines were declared under the numbers DC2008-585 and DC2011-1437 to the Ministry of Research. The database derived from the COPERNIC collection is protected under the reference IDDN.FR.001.510017.000.D.P.2014.000.10300.

All the anonymous patients were informed and signed consent according to the ethics recommendations. This collection is composed of cancer patients presenting with overreactions in normal tissues after radiotherapy. Severity of side-effects was graded for each patient according to the Common Terminology Criteria for Adverse Events scale, version 4.03 (17). Sampling was performed in non-irradiated, non-photo-exposed anatomical region after local anesthesia. Standardized dermatological punch and untransformed fibroblast cell strains were prepared from skin biopsies. In the present study, 16 breast cancer patient cells were studied, comprising eight cell strains from grade 2 patients, here referenced as P1 to P8, and eight cell strains from grade 3 patients (P9 to P16). Cells were studied between passage 7 to 10 in culture (mean population doublings: 35 to 50), before any senescence occurrence.

As control, primary dermal fibroblasts were obtained from eight non-irradiated female healthy donors (C1 to C8). Surgical samples were obtained from the Hospitals Board of Lyon, France (Hospices Civils de Lyon), with the subjects’ informed consent. Cells were subcultured up to seven passages and studied between passage 7 to 10, to have similar age in culture as patient cells (mean population doublings: 35 to 50). As fibroblasts are quiescent cells in the dermis, most studies were performed on confluent cells, in the G0/G1 cell cycle phase, both for patient and control cells.

Cultures were maintained in Dulbecco’s Modified Eagle Medium–Glutamax medium (ThermoFisher Scientific, Illkirch, France) supplemented with 10% fetal bovine serum (ThermoFisher) and 1% penicillin/streptomycin (Sigma-Aldrich, Saint-Quentin-Fallavier, France).

### Cell Irradiation

Primary dermal fibroblasts were irradiated after reaching confluency with 2 Gy using an XRAD320 X-ray generator (Precision X-Ray, North Brandford, CT, USA) at a dose rate of 0.8 Gy.min^−1^ and then further cultured for indicated times depending on the assay.

### Colony Survival Assay

Dermal fibroblasts were irradiated with 2 Gy X-rays after reaching confluency and seeded at low density (5 to 40 cells/cm²) 24 h after irradiation. Two weeks later, cell cultures were fixed with EtOH 100% for 15 min and stained with hematoxylin/eosin. Only colonies formed by more than 50 fibroblasts were considered for calculating survival fraction at 2 Gy (SF2), expressed as the ratio between colonies formed with and without irradiation.

### γH2AX and 53BP1 Foci Assays

Irradiated fibroblasts were further cultured for the indicated times (0, 15 min, 2, 6, and 24 h) and fixed with 4% paraformaldehyde for 15 min. Then, cells were permeabilized (0.1% Triton X-100 and 0.1 M Glycine) and incubated in blocking buffer (5% goat serum, 2% BSA, 0.1% Triton X-100, and 0.05% Tween-20) for 15 min prior to immunostaining with anti-γH2AX antibody (05-636, Millipore) or anti-53BP1 antibody (PA1-46147, ThermoFisher). For immunodetection, goat anti-rabbit IgG or goat anti-mouse IgG Alexa Fluor-488 or -546 conjugated secondary antibody (ThermoFisher) was incubated for 1 h and nuclei were counterstained with 4′-6-diamidino-2-phenylindole dihydrochloride (DAPI). The resulting foci were counted in at least 50 nuclei per condition using an Eclipse Ti-E inverted microscope (Nikon).

### DNA Repair Chip Assay

Fibroblast DNA repair was measured on ExSy-SPOT assay (LXRepair). Protein extracts from lysed cells were applied directly on the biochip containing plasmids with well-characterized DNA lesions (8-Oxoguanine, Ethenobase, Abasic site, Glycols, Photoproducts and Cisplatin adducts) and incubated with fluorescent nucleotides to allow DNA repair. Effective DNA repair was quantified for each type of lesion by CT measurement of the resulting fluorescence signal. Thus, fluorescence level was proportional to cell ability to repair the specific DNA damage within the prescribed time.

### Transcriptome Analysis

Total RNA was isolated from fibroblasts at confluency with the NucleoSpin RNA plus kit (Macherey–Nagel, Hoerdt, France) or RNeasy Plus Minikit (Qiagen, Courtaboeuf, France) according to the manufacturer’s instructions.

For next-generation RNA sequencing at CNRGH (CEA, Evry, France), RNA sequences were captured using a TruSeq RNA Library Prep Kit v2 (Illumina, Evry, France) with input of 1 µg. Paired-end RNA sequencing was performed on HiSeq4000 with 100 bp paired-end reads. Sequencing data quality control was performed using FastQC 0.11.7 before and after adapter trimming by Cutadapt 1.13 (parameters: -q 15 -a 5’-AGA TCG GAA GAG CAC ACG TCT GAA CTC CAG TCA C-3’ – 5-AAG ATC GGA AGA GCG TCG TGT AGG GAA AGA GTG TAG ATC TCG GTG GTC GCC GTA TCA TT-3’). The reads were mapped to the human genome (GRCh37/hg19) using HISAT2 2.0.5. For a single gene, sequences were aligned *versus* all known exons of all gene isoforms. The resulting BAM files were sorted by read pairs (using SAMtools 1.3.1) and counted using the HtSeq-count tool of HtSeq 0.6.1.

For transcriptome analysis, filtering was applied to genes with low expression (mean number of reads in the training set <10). Principal component analysis and hierarchical clustering were performed using the R DEseq2 and stats packages. VennDiagram and plots were made using the VennDiagram and ggplot2 packages. Functional annotation of the gene list was performed using the WEB-based GEne SeT AnaLysis Toolkit (WebGestalt) and the KEGG database 2019. Gene Ontology (GO) was performed using the clusterProfiler package in R software (18). GO and KEGG enrichment analyses were based on a false discovery rate (FDR) threshold of <0.05. The Enrichedplot package was used for graphical visualization of the result from enriched analysis. Transcriptome data have been deposited into the GEO database and are available under the accession number GSE154559.

### Real-Time Quantitative PCR

An equal amount of total RNA (500 ng) was used as template for reverse transcription with PrimeScriptTM RT reagent kit (Takara, Shiga, Japan) and analyzed by Real-Time QPCR using SYBR^®^ Premix ExTaqII (Takara) on an AriaMx Realtime PCR system (Agilent Genomics, Santa Clara, CA, USA). All primers listed below were provided by Eurogentec. *NFATC2* expression level was normalized to *TBP* and *RPS17* housekeeping gene expression level.

*NFATC2f:* 5’-TTGGAAGAAAGAACACGCGG-3’,
*NFATC2r:* 5’-GAGCACTCGATGGGGTTAGA-3’,
*TBPf*: 5’-TCAAACCCAGAATTGTTCTCCTTAT-3’,
*TBPr:* 5’-CCTGAATCCCTTTAGAATAGGGTAGA-3’,
*RPS17f:* 5’-CTCTTTTACCAAGGACCCGCC-3’,
*RPS17r:* 5’-AGGTTGGACAGACTGCCGAAG-3’

### Protein Extraction and Immunoblotting

Total proteins were extracted using RIPA buffer (50 mM Tris-HCl pH = 8, 150 mM NaCl, 1.5 mM KCl, 1% NP-40, 0.1% SDS, 0.5% sodium deoxycholate, 0.1% Triton X-100, 1 mM EDTA) containing protease inhibitor cocktail (cOmplete mini, Roche Diagnostics) and phosphatase inhibitor cocktail (5 mM NaF, 50 mM β-glycerophosphate, 5 mM orthovanadate). Proteins were quantified using the Pierce BCA Protein Assay Kit (ThermoFisher), loaded on an 8% SDS-polyacrylamide gel and transferred to a nitrocellulose membrane (Bio-rad). The membrane was blocked for 1 h at room temperature in TBS-Tween20 0.1–5% BSA and immunoblotted overnight at 4°C for primary antibodies specific to NFATC2 (#4389, Cell Signaling Technology) or VINCULIN (V9131, Sigma-Aldrich). After washing, goat anti-mouse IgG or goat anti-rabbit IgG HRP-conjugated secondary antibodies (Bio-rad) were incubated for 1 h at room temperature. Proteins were revealed using SuperSignal West Pico PLUS Chemiluminescent Substrate (ThermoFisher) and the signal was detected by the Fusion Fx system (Vilber Smart Imaging). Immunoblot quantifications were performed using GelAnalyzer software.

### DNA Extraction and Bisulfite Conversion and HRM PCR

DNA was extracted from fibroblasts using the QIAamp DNA Mini Kit (Qiagen) according to the manufacturer’s instructions. To assess methylation of a specific DNA region, DNA was converted with bisulfite treatment, using the EpiTect Bisulfite Kit (Qiagen) according to the manufacturer’s instructions. Then, HRM PCR was performed using the EpiTect HRM PCR kit (Qiagen) to amplify the specific DNA region and to measure the melting temperature of the amplicon on an AriaMx Realtime PCR system (Agilent Genomics). One hundred percent methylated DNA, 100% unmethylated DNA, and bisulfite unconverted DNA from the EpiTect Control DNA Set kit (Qiagen) were used as controls. Figures with methylation peaks were produced with Agilent Aria 1.5 Software (Agilent Genomics). Primers were designed with Methyl Primer Express Software v1.0 (ThermoFisher Scientific) and were provided by Eurogentec. The following primers were used for methylation study:

*NFATC2mF*: 5’-TTTAGATGAATAGTGTTTTGGG-3’,
*NFATC2mR*: 5’-ATTATCATTTCCTTCCTCTACTTC-3’.

### RNA Interference

Control fibroblasts were transduced with lentiviral vectors from NFATC2 Human shRNA Plasmid Kit (OriGene). Lentiviral vector particles were produced by the vector facility at SFR BioSciences Gerland-Lyon Sud (Lyon, France) as previously described (19). Control cells were infected at 40% confluency with lentiviral particles (MOI at 10) containing a vector with a shRNA targeting *NFATC2* (sh-*NFATC2*) or a plasmid with a non-effective shRNA sequence (sh-*SCR*) for 12 h. At confluency, cells were trypsinized and seeded in another plate. Then, transduced cells were maintained under puromycin selection for 1 week and then selected cells were amplified for 1 week before analysis.

### Statistics

Statistical significance was calculated by Student’s t-test, one-way analysis of variance (ANOVA), two-way analysis of variance (ANOVA2), or Pearson correlation using Prism software (version 8.0, GraphPad Software). Mean differences were considered statistically significant when *P* < 0.05. * *P* < 0.05, ** *P* < 0.01, *** *P* < 0.001, **** *P* < 0.0001.

## Results

### Fibroblasts From Patients With Severe Radiotherapy Side Effects Exhibit Decreased Tolerance to Radiation Toxicity

**Figure 1:**
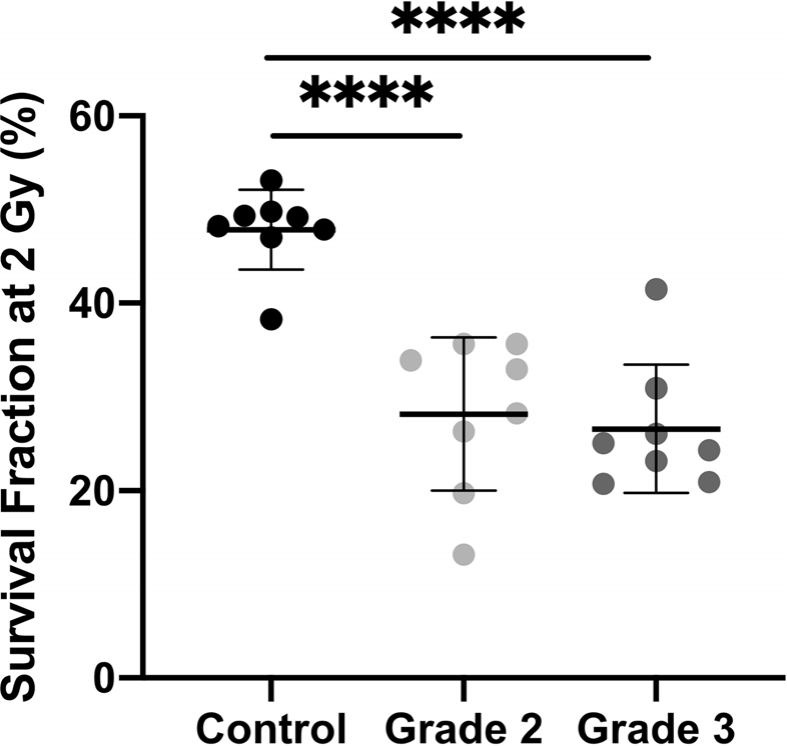
Dermal fibroblasts from patients with severe side-effects of radiotherapy exhibit higher radiosensitivity. Colony survival assays showed that survival fraction at 2 Gy was higher in control cells (n = 8) than in cells from overreacting patients, with no difference according to clinical grade (n = 8 grade 2 and n = 8 grade 3). Results are mean +/− SD. The p-value was calculated by one-way ANOVA. Significant at ****P < 0.0001.

We analyzed ionizing radiation toxicity in cultured dermal fibroblasts from sixteen breast cancers patients with severe radiotherapy (RT) side-effects (eight grade 2, eight grade 3) and eight control individuals. To estimate radiation-induced toxicity, we used the reference method of colony survival fraction measurement after a standard dose of 2 Gy X-irradiation (SF2). The colony survival assay showed that dermal fibroblasts from the eight control biopsies presented a mean 48% survival fraction, whereas cells from patients with RT over-reaction exhibited significantly lower SF2, with 28 and 27% survival fraction for cells of grade 2 and grade 3 patients respectively (**Figure 1**). Since no significant difference in cellular radiosensitivity was observed between cells from grade 2 and grade 3 patients, the 16 cell strains were pooled for the following experiments.

### Fibroblasts From Patients With Severe Radiotherapy Side Effects Exhibit DNA Repair Defects

**Figure 2:**
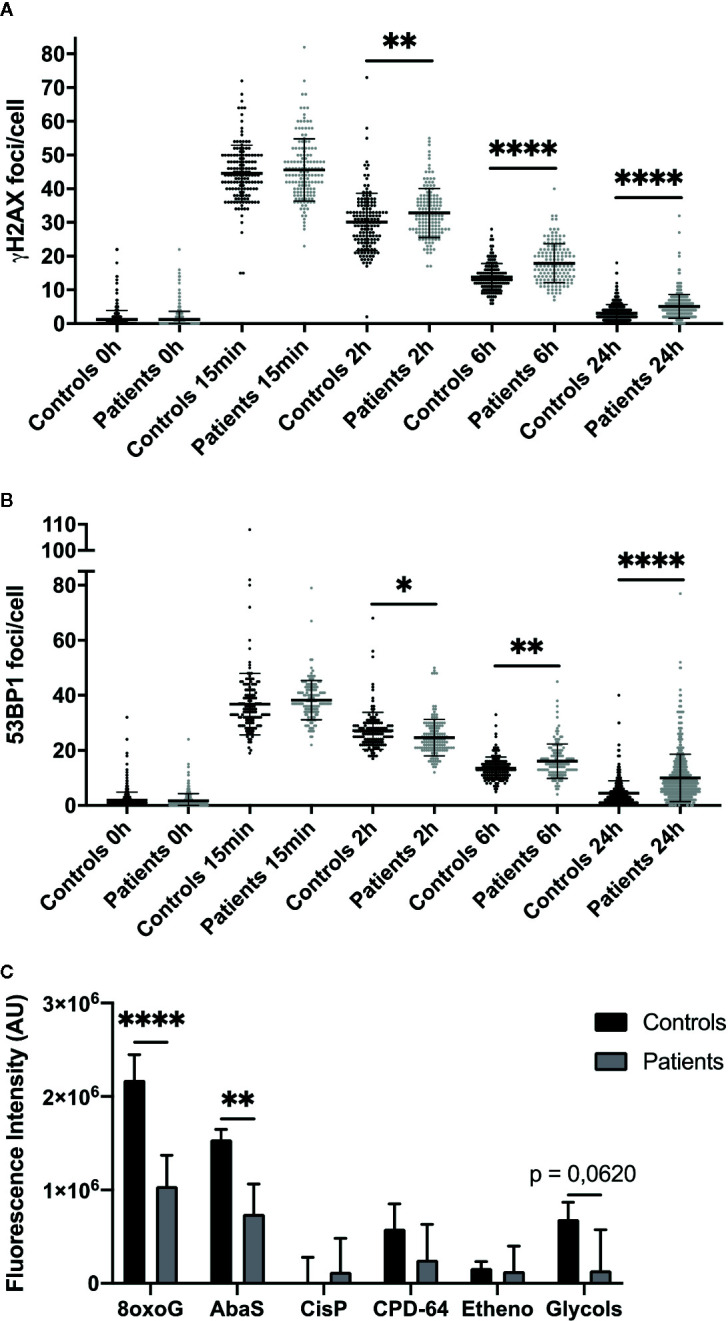
Impaired DNA repair in dermal fibroblasts from overreacting patients. Immunofluorescence detection of γH2AX **(A)** and 53BP1 **(B)** foci, investigated 0 h, 15 min, 2, 6, and 24 h after 2 Gy irradiation in cells from patients with severe radiotherapy side-effects. The number of foci was assessed in at least 100 cells in four normal (C1, C2, C4, and C8) and four patient cell strains (P1, P6, P10, and P15). Results are mean +/− SD. The p-value was calculated by one-way ANOVA. Significant at *P < 0.05, **P < 0.01, and ****P < 0.0001. **(C)** DNA damage repair ability was measured using the ExSy-SPOT chip. Fluorescence was proportional to cell ability to repair indicated DNA lesions. 8oxoG, 8-oxoGuanine; AbaS, Abasic site; CisP, Cisplatin adducts; CPD-64, Cyclobutane pyrimidine dimer - pyrimidine- (6,4)-pyrimidone photoproducts; Etheno, Etheno adducts. Results are mean +/− SD from four control fibroblast strains (C1, C2, C4, and C6) and four radiosensitive fibroblast strains (P2, P7, P8, and P10). The p-value was calculated by Student’s t-test. Significant at **P < 0.01 and ****P < 0.0001.

To study patient cell DNA repair ability, we first performed immunofluorescence against γH2AX and 53BP1, two early markers of DNA double-strand breaks (DSB), in four control and four radiosensitive cell strains after 2 Gy irradiation. The number of γH2AX and 53BP1 foci 15 min after 2 Gy irradiation was identical in control and patient fibroblasts, with a mean 40 foci per nucleus in both (**Figures 2A, B**). However, more γH2AX foci were detected in patient than control cells 2, 6, and 24 h after irradiation: 2.7, 4.13, and 2.03 additional foci per cell respectively (**Figure 2A**). More 53BP1 foci were also detected 6 and 24 h after 2 Gy irradiation in patient than control fibroblasts: respectively +2.65 and +5.55 foci per cell (**Figure 2B**).

We also assessed patient cell ability to repair various DNA lesions, using the ExSy-SPOT chip, a microsystem developed to measure excision-synthesis activity in immobilized plasmid DNA (20). Repair activity was reflected by the incorporation of fluorescent nucleotides at the lesion site. Fluorescence in plasmids containing 8-oxoGuanine and abasic sites was higher in control than patient samples (**Figure 2C**), and in plasmids containing glycol-damaged bases, although the difference did not quite reach significance (*P* = 0.062) (**Figure 2C**). Thus, ability to repair 8-oxoGuanine, abasic site and glycol-damaged bases, three types of DNA damage induced by oxidative stress and known to be repaired by the BER pathway, seemed to be impaired in dermal fibroblasts from patients with severe radiotherapy side-effects.

### Identification of a Specific Transcriptome Profile in Patients’ Fibroblasts

**Figure 3:**
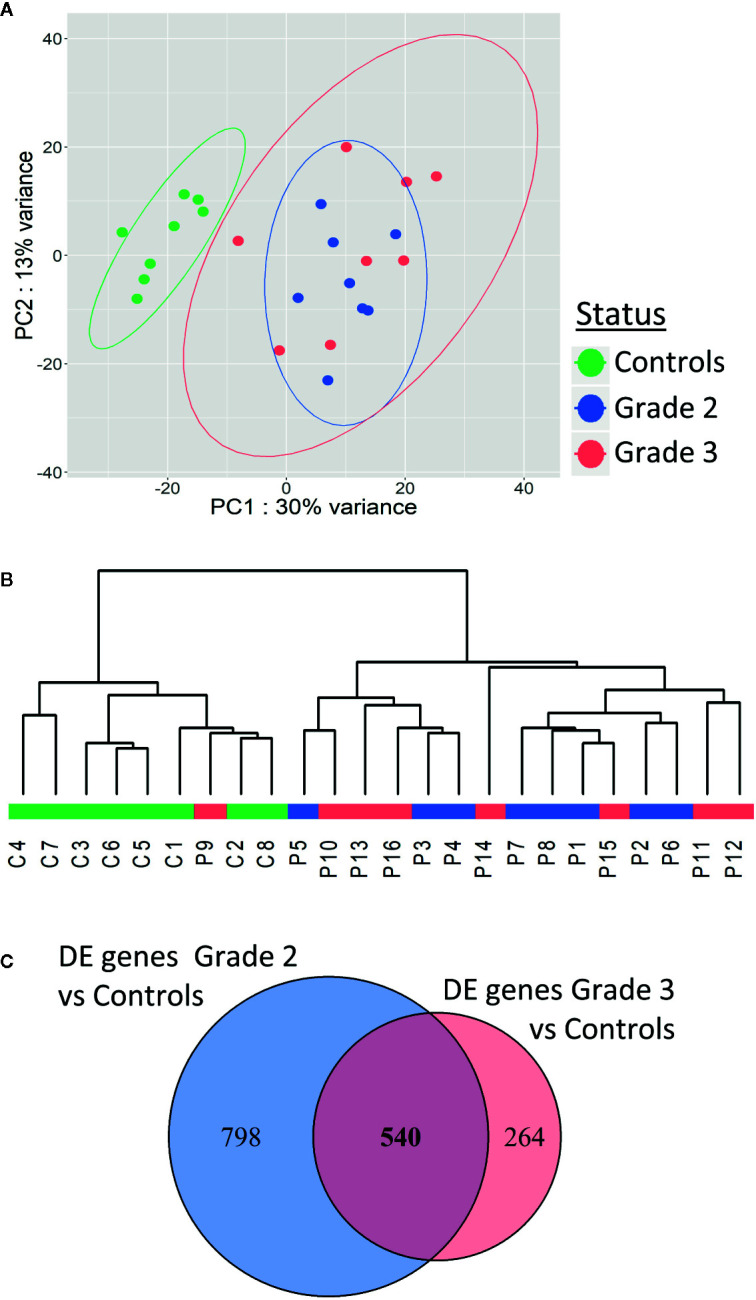
Patients with severe radiotherapy side-effects show a specific transcriptome profile. Principal component analysis **(A)** and hierarchical clustering **(B)** of patients and controls based on RNA sequencing data. **(C)** Venn diagram showing the 540 differentially expressed genes in common between grade 2 vs control and grade 3 vs control RNAseq data.

To investigate the mechanisms underlying individual radiosensitivity at cellular level, we used next-generation RNA sequencing to profile the whole genome transcriptome of the 16 patient and 8 control fibroblast cultures. Principal component analysis (PCA) of whole gene expression data clearly separated controls from over-reacting patients (**Figure 3A**). However, one patient’s cell strain was classified as being in the control group by hierarchical cluster analysis (**Figure 3B**). Within the patient group, there was no clear separation between grades 2 and 3, whether on PCA or hierarchical cluster analysis (**Figures 3A, B**).

We identified 1,338 genes differentially expressed (adjusted p-value <0.05) between fibroblasts from grade 2 patients and controls (**Figure 3C**), and 804 genes differentially expressed between fibroblasts from grade 3 patients and controls (**Figure 3C**). Five hundred forty of these differentially expressed genes were in common between grade 2 patients *vs* controls and grade 3 patients *vs* controls (**Figure 3C** and **Supplementary Table S1**). Interestingly, no genes were differentially expressed between grade 2 and grade 3 patients, in agreement with the PCA and hierarchical cluster classification of the transcriptome data.

Four hundred forty-five of the 540 differentially expressed genes were protein-coding and were used for functional enrichment analysis. Gene ontology analysis highlighted 93 significantly enriched GO biological processes with FDR <0.05 (**Figure S1 Supplementary data**). Functions with the smallest FDRs comprised regulation of GTPase activity regulation, of organ development and of cell adhesion and junction (**Figure S1 Supplementary data**). All these functions might be involved in cellular radiation toxicity, but correspond to multiple intracellular pathways. We then searched for enriched pathways involving the 445 differentially expressed protein-coding genes using the WebGestalt and the KEGG database 2019. We found only one significantly enriched pathway (**Supplementary Table S2**): Arrhythmogenic right ventricular cardiomyopathy (adjusted p-value 0.0065733—10 genes out 72 modulated), a pathology of cardiac muscles with progressive loss of myocytes replaced by adipocytes. Systematic analysis of our gene list revealed that none of these genes was directly involved in any known genetic syndrome leading to increased radiosensitivity.

### NFATC2 Is Downregulated in Patient Cells and Correlated With Radiosensitivity

**Figure 4:**
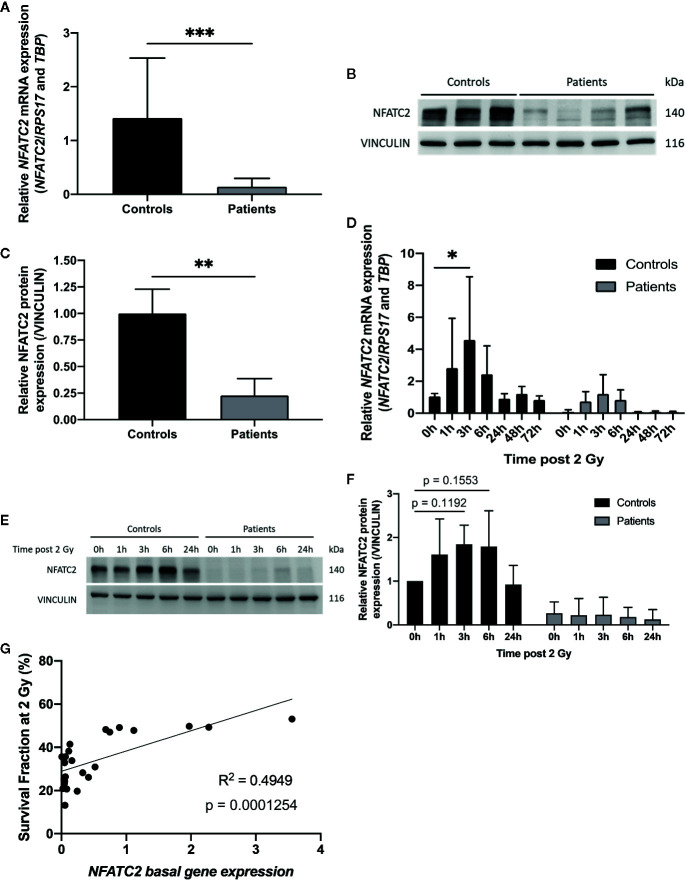
NFATC2 downregulation in fibroblasts from overreacting patients. **(A)** *NFATC2* mRNA levels were measured by RTqPCR in control cells (n = 8) and in fibroblasts from patients with severe radiotherapy side-effects (n = 16). Results are mean +/− SD. The p-value was calculated by Student’s t-test. Significant at ***P < 0.001. **(B)** NFATC2 protein expression was evaluated by immunoblotting in control (n = 3) and patient fibroblasts (n = 4) and quantified **(C)**, with VINCULIN as loading control. Results are mean +/− SD. The p-value was calculated by Student’s t-test. Significant at **P < 0.01. **(D)** *NFATC2* gene expression was assessed at various time points after 2 Gy irradiation in three control cell strains (C1, C5, and C8) and three cell strains from overreacting patients (P7, P8, and P10). Results are mean +/− SD. The p-value was calculated by two-way ANOVA. Significant at *P < 0.05. **(E)** Representative image of immunoblotting analysis of NFATC2 protein expression at various time points after 2 Gy irradiation on one control cell strain (C4) and one patient cell strain (P8) and quantification **(F)**. Results are mean +/− SD from three independent immunoblotting analyses of three control (C4, C5, and C8) and three patient fibroblast strains (P6, P8, and P10). The p-value was calculated by two-way ANOVA. **(G)** Pearson correlation between *NFATC2* gene expression and SF2.

Among the most differentially expressed genes between control and patient fibroblasts identified by our RNA sequencing approach, we focused on *NFATC2*, which encodes a transcription factor initially described in T-cell activation and involved in numerous cellular functions such as apoptosis and the cell cycle (21). We therefore carefully analyzed NFATC2 gene and protein expression in patient cells extracted from non-irradiated skin. Dermal fibroblasts from patients with severe radiotherapy side-effects exhibited much lower *NFATC2* gene expression than control cells (**Figure 4A**). Similarly, NFATC2 protein expression was lower in patient’s cells (**Figures 4B, C**). NFATC2 gene and protein expressions were assessed after irradiation to determine whether *NFATC2* could be a radiation-responding gene, potentially involved in cellular radiation response. *NFATC2* gene overexpression was detected in response to 2 Gy irradiation, with a 4-fold peak at 3 h in control fibroblasts and a 15-fold peak in patient fibroblasts, with return to baseline after 24 h (**Figure 4D**). NFATC2 protein was detected in greater quantities (×1.8) after 2 Gy irradiation with a peak between 3 and 6 h in normal fibroblasts, but the difference was not statistically significant due to interindividual variability (*P* = 0.1192 and *P* = 0.1553, respectively) (**Figures 4E, F**). Furthermore, NFATC2 protein was barely detected in cells from radiosensitive patients (**Figures 4E, F**). Interestingly, there was a significant correlation between NFATC2 transcriptional expression and irradiated cell survival (Pearson correlation coefficient, R² = 0.4949, P = 0.0001254) (**Figure 4G**), suggesting a possible role for NFATC2 in cellular radiosensitivity. Taken together, these results suggest that *NAFTC2* is a radiation-responding gene potentially involved in cellular response to ionizing radiation.

### *NFATC2* Is Hypermethylated in Patient Fibroblasts

**Figure 5:**
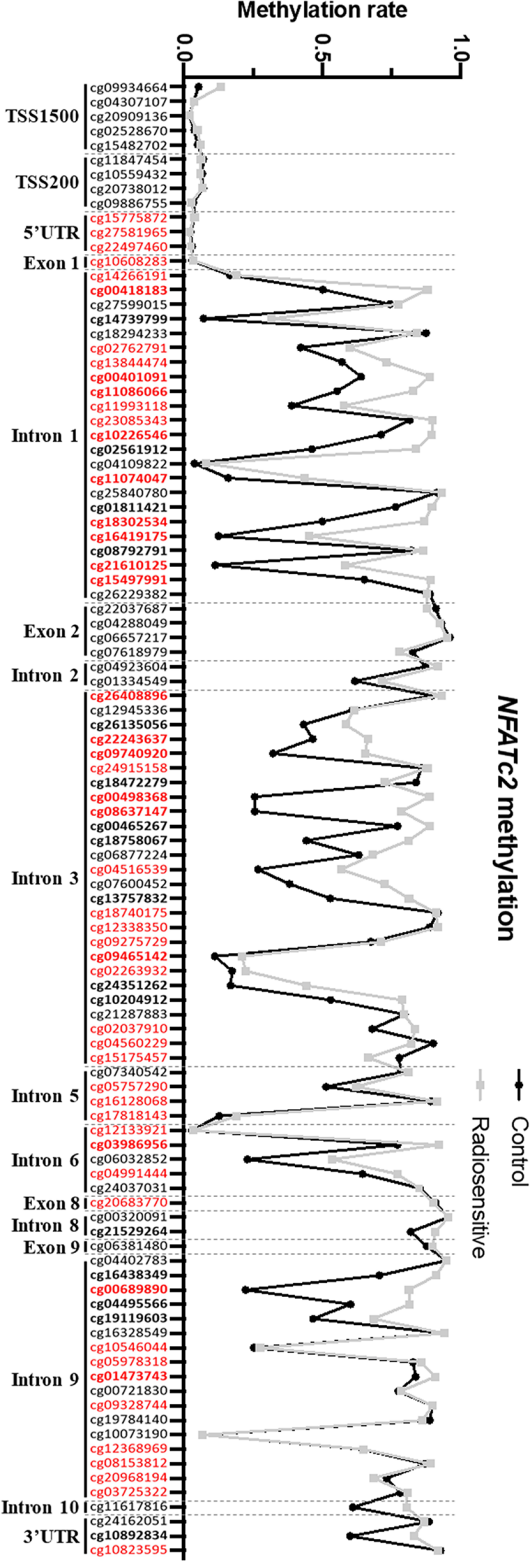
*NFATC2* is hypermethylated in patient fibroblasts. *NFATC2* methylation in cells from patients with severe radiotherapy side-effects (n = 16) and controls (n = 8) were investigated on genome-wide methylation analysis. CpG sites in bold are differentially methylated between control and patient cells. CpG sites in red are part of promoter-associated regions defined by the ENCODE consortium.

To elucidate the regulatory mechanisms underlying *NFATC2* differential expression in non-irradiated fibroblasts from controls and patients, we compared gene methylation level between patients and controls. The methylation pattern of the *NFATC2* gene region was extracted from genome-wide methylation profiling using methylation bead chips performed on patient and control fibroblasts (data not shown), identifying 34 differentially methylated sites (CpGs) (33 hyper- and 1 hypo-methylated) (**Figure 5**). This hypermethylation of the *NFATC2* gene in patients’ fibroblasts was consistent with its lower gene expression. Interestingly, the upstream region of the transcription starting site (TSS) exhibited the same methylation profile in patient and control cells (**Figure 5**). However, among the hypermethylated CpGs, cg00418183, cg00401091, cg11086066, cg10226546, cg11074047, cg18302534, cg16419175, cg21610125, cg15497991, cg26408896, cg22243637, cg09740920, cg00498368, cg08637147, cg09465142, cg03986956, and cg00689890 belong to gene regions identified as promoter-associated regions according to the ENCODE consortium. This hypermethylation of these regulatory regions could, at least in part, explain the weak expression of *NFATC2* in patient fibroblasts.

To confirm these results by an independent method, we performed HRM PCR to evaluate the methylation state of the CpG00498368 in four patient and two control cell strains. HRM PCR is a PCR measuring the melting temperature of a specific amplicon. After bisulfite conversion, an amplicon comprising a methylated CpG would exhibit a higher melting temperature than an amplicon with an unmethylated CpG. We detected a higher melting temperature peak, corresponding to a methylated state of this CpG in patient fibroblasts and to an unmethylated state in control fibroblasts (**Figure S2B Supplementary data**), in agreement with the global methylome data (**Figure S2A Supplementary data**).

These results suggest that NFATC2 down-regulation could, at least in part, be due to hypermethylation of the gene in fibroblasts from patients with severe radiotherapy side-effects.

### NFATC2 Silencing in Normal Dermal Fibroblasts Leads to Increased Cellular Radiosensitivity and DNA DSB Defects

**Figure 6:**
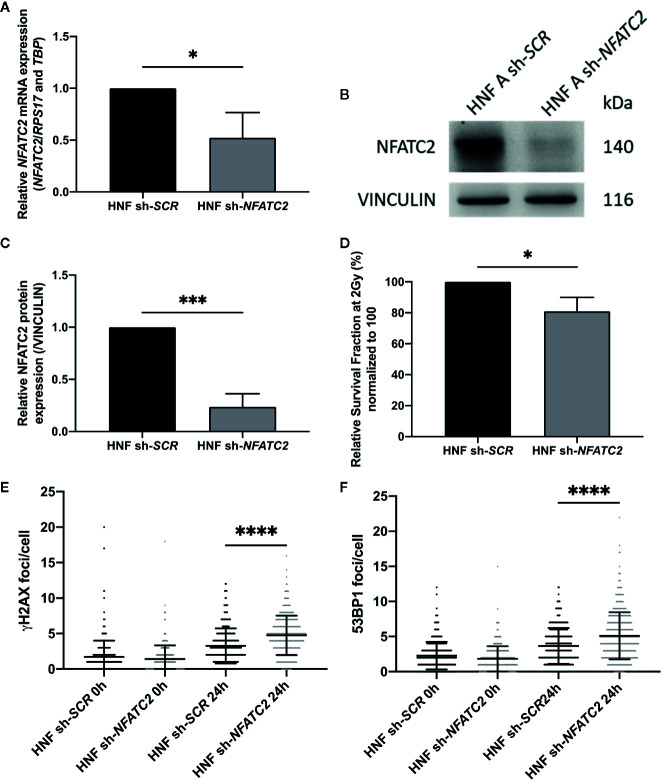
NFATC2 downregulation leads to cellular radiosensitivity. **(A)** *NFATC2* mRNA levels were measured by RT-qPCR in three control fibroblast strains (HNF) infected with a lentiviral vector carrying either a shRNA scramble (sh-*SCR*) sequence or a shRNA targeting *NFATC2* (sh-*NFATC2*). Results are mean +/− SD. The p-value was calculated using a Student’s t-test. Significant at *P < 0.05. **(B)** Representative image of immunoblotting analysis of NFATC2 protein expression in one control cell strain (HNF A) infected with lentiviral vectors sh-*SCR* or sh-*NFATC2*, and quantification **(C)**. Results are mean +/− SD from immunoblotting analysis of three different cell strains infected with lentiviral vectors sh-*SCR* or sh-*NFATC2*. The p-value was calculated using a Student’s t-test. Significant at ***P < 0.001. **(D)** Lower SF2 was measured by colony survival assays in cells infected with lentiviral vector sh-*NFATC2* compared to cells infected with lentiviral vector sh-*SCR*. Results are mean +/− SD from three cell strains. The p-value was calculated using a Student’s t-test. Significant at *P < 0.05. More numerous γH2AX **(E)** and 53BP1 **(F)** residual foci, investigated by immunofluorescence, 24 h after 2 Gy irradiation in fibroblasts infected with lentiviral vector sh-*NFATC2*. Assessed in at least 100 cells in three cell strains infected with lentiviral vectors sh-*SCR* or sh-*NFATC2*. Results are mean +/− SD. The p-value was calculated using a two-way ANOVA. Significant at ****P < 0.0001.

To evaluate the functional impact of NFATC2 in cellular radiosensitivity, *NFATC2* expression was silenced in cells from healthy individuals by stable RNA interference mediated by a lentiviral vector. We tested four lentiviral vectors, each containing a different short hairpin RNA (shRNA) targeting *NFATC2*, and chose the most efficient in terms of silencing for the further experiments (**Figure S3 Supplementary data**). NFATC2 expression was reduced by 48% on average at gene level (**Figure 6A**) and by 70% on average at protein level in transduced fibroblasts expressing the shRNA targeting the *NFATC2* transcript (**Figures 6B, C**). Colony survival assays revealed that cells with lower *NFATC2* expression (HNF sh-*NFATC2*) exhibited a significantly lower SF2 compared to their control (HNF sh-*SCR*) (20% decrease in cell survival in response to irradiation) (**Figure 6D**). To better understand the molecular mechanisms of this cell death elevation in sh-NFTAC2 expressing cells, we investigated their DNA DSB repair ability, and detected more residual γH2AX and 53BP1 foci 24 h after 2 Gy irradiation in HNF sh-*NFATC2* cells than in control cells (HNF sh-*SCR*) (respectively, +1.49 γH2AX and +1.46 53BP1 foci per nucleus on average) (**Figures 6E, F**), corresponding to an excess of residual unrepaired DNA double-strand breaks. These results suggest that the *NFACT2* down-regulation observed in patient cells is involved in their cellular radiation sensitivity.

## Discussion

The mechanisms responsible for individual sensitivity to ionizing radiations are not yet fully elucidated. The present study investigated the radiation sensitivity of dermal fibroblasts from patients showing severe side-effects of radiotherapy (RT). A key result was that patient dermal fibroblasts were intrinsically more radiosensitive than cells from healthy individuals. We also detected more γH2AX and 53BP1 foci from 6 to 24 h after irradiation in patient dermal fibroblasts, suggesting slower DNA DSB repair ability.

DNA DSB repair abilities in cells from radiosensitive patients have been widely studied. Some authors identified a link between residual unrepaired DNA DSB and risk of severe RT side-effects (16, 22, 23), whereas others found no DNA DSB repair defect in cells from radiosensitive patients (24–26). Once again, these differences could be due to differences in the methods used to assess DSB repair ability, highlighting the necessity of more standardized protocols, especially regarding cell culture conditions, type of irradiation and DSB detection kinetics (27).

Moreover, using the ExSy-SPOT assay, we showed that cells from patients with severe RT side-effects also exhibited repair defects for 8-oxoGuanine, abasic sites, and glycol-damaged bases. All these types of DNA damage, induced by ionizing radiation and the resulting oxidative stress, are usually repaired by base excision repair mechanisms (28). Batar et al. showed that a decrease in *XRCC1* expression, an actor in base excision repair, was associated with acute side-effects in breast cancer patients (29). However, no *XRCC1* differential expression was detected between control and patient fibroblasts and further investigations will be needed to elucidate at which level dermal fibroblasts from radiosensitive patients fail in their base excision repair mechanism.

Patient cells exhibited a specific transcriptome profile compared to controls, with no segregation at transcriptional level between clinical grades 2 and 3. Side-effect severity is probably highly multifactorial and modulated by complex interaction between intrinsic and extrinsic factors during radiotherapy treatment. This could explain why we were not able to distinguish clinical grades on transcriptome analysis of patients’ cells obtained several months after end of treatment. Nevertheless, we were able to identify 540 genes differentially expressed between dermal fibroblasts from grade 2 and 3 patients *versus* controls. This list of genes is a potentially valuable resource for identifying new modulators of radiation toxicity in tissues and cells. Surprisingly, only one biological process, arrhythmogenic right ventricular cardiomyopathy (ARVC), was significantly over-represented in this gene list. ARVC is a non-genetic disease without any evidence of associated radiosensitivity. The link between ARVC and individual radiation response seems to be incidental, but it has been shown that inhibition of the Wnt pathway is a causal mechanism in ARVC (30), and it is clearly established that the Wnt pathway plays an important role in cell survival after irradiation, as previously shown in several cell types (31–33).

The main limitation of this transcriptome study is the relatively small number of patients analyzed, with 16 patients who suffered severe RT side-effects and 8 control samples. Nevertheless, this study allowed us to identify 540 genes differentially expressed between controls and patients, including NFATC2. We recently analyzed the expression of *NFATC2* in dermal fibroblasts from 22 additional patients and 8 controls and confirmed the strong repression of this gene in the patient’s cells (fold change 4.07, p-value 3.21 E-1).

Transcriptome analysis identified *NFATC2* as one of the genes most differentially expressed between cells from over-reacting patients and controls. We confirmed that NFATC2 expression at gene and protein levels at basal state was lower in fibroblasts from patients with severe RT side-effects, and that *NFATC2* gene expression was modulated in response to irradiation. Furthermore, we highlighted the global hypermethylated state of *NFATC2* in patient fibroblasts, suggesting a role of methylation in the regulation of NFATC2 expression. Particularly, 17 CpG sites located in the promoter-associated regions defined by the ENCODE consortium (34) were identified as hypermethylated in patient fibroblasts. These promoter-associated regions were identified by ChIP-seq analysis against transcription factors in 91 different cell lines. Several transcription factors were identified by the ENCODE consortium as able to recognize the DNA region where the 17 CpG sites are located, including CTCF, E2F6, ZBTB7A, ZNF143, JUND, MEF2A, EGR1, RUNX3, and EBF1. ChIP analysis against these transcription factors in irradiated fibroblasts and at basal state would be of interest to elucidate the regulatory mechanisms of *NFATC2* expression modulated by epigenetic mechanisms.

Moreover, the present study showed that *NFATC2* silencing using RNA interference leads to increased cellular radiosensitivity and to a defect in DNA DSB repair. To our knowledge, this was the first study linking NFATC2 and cellular sensitivity to ionizing radiation. NFAT family members have been shown to be able to induce *GJA1* and *GADD45A* gene expressions in response to ionizing radiation (35, 36) and NFATC2 has been shown to induce *IL-5* expression in response to ionizing radiation (37). However, NFAT response to UV irradiation has been more thoroughly studied. It has been shown that NFAT positively regulates apoptosis in response to UV-radiation in keratinocytes (38) but, in contrast, inhibiting NFAT signaling promoted apoptosis in response to UV irradiation in a human embryonic fibroblast cell line (39). Moreover, inhibiting NFAT signaling has been shown to reduce keratinocyte ability to repair UV-induced DNA damages (40, 41). However, these studies used chemical inhibitors of NFAT signaling, while the specific roles of each NFAT family member in response to radiation remain unknown.

NFATC2 has been shown to regulate apoptosis and cell cycle progression, two major mechanisms involved in cellular radiosensitivity. NFATC2 controls the expression of *FASLG*, a pro-apoptotic regulator (42, 43), and of *CFLAR*, *BCL2A1*, and *MDM2*, known for their anti-apoptotic abilities (44–46). Moreover, NFATC2 can also regulate *TNFA* and *NR4A1* expression, both known for their dual pro- and anti-apoptotic roles (47, 48). Furthermore, NFATC2 has been shown to regulate cell cycle progression positively by inducing expression of *CDK6* (49) or inhibiting expression of *CDK4* (50), or negatively by inducing expression of *CDKN1A* (51) or repressing expression of *CCNA2* (52) and *p15^INK4b^* (53). NFATC2 has also been reported in positive or negative regulation of *MYC* expression, promoting or blocking cell cycle progression (54). These dual roles of NFATC2 in apoptosis and cell cycle control highlight the importance of its different isoforms and partners. One limit of our functional study of NFATC2 is the use of a shRNA targeting a region common to all the known isoforms of this protein. Further experiments using isoform-specific shRNAs will be necessary to decipher the relative role of the different variants of NFATC2 in fibroblast sensitivity to ionizing radiations.

In a 2016 study, Gabriel et al. investigated NFATC2 isoform C partners in a Jurkat human T-cell line (55). In addition to transcription factors, the authors pointed out new potential NFATC2 partners, including several actors of DNA damage response: notably those involved in DNA DSB repair, such as XRCC5/Ku80 and PRKDC, and in base or nucleotide excision repair, such as RFC, LIG3, and XRCC1. They also found an interaction between NFATC2 and PARP1, which is involved in recognition of DNA damage, and RPA, which binds to single-strand DNA during DNA repair. Interestingly, the authors detected these associations between actors of DNA damage response and NFATC2, but not with NFATC1, which suggests a specific role for NFATC2 in the DNA damage response (55). We performed preliminary experiments to clarify the interactions between NFATC2 and a few DNA repair proteins such as XRCC1 and XRCC5 in irradiated and non-irradiated fibroblasts, but did not observe any consistent co-immunoprecipitation (data not shown). Further specific investigations will be necessary to clarify these putative interactions, using tagged versions of the NFATC2 protein.

The present study found increased cellular radiosensitivity and a defect in DNA repair in dermal fibroblasts from patients with severe side-effects of RT, and highlighted a specific transcriptome profile in patient fibroblasts. These data pave the way for cellular and molecular strategies to identify radiosensitive patients. The study also showed the involvement of NFATC2 in cellular sensitivity to ionizing radiation and in DNA repair. However, the mechanisms by which NFATC2 contributes to the cellular response to ionizing radiation remain to be clarified, notably concerning its interactions with actors of the DNA damage response, and its target genes as transcription factor. For the latter, investigating molecular pathways known to be involved in the development of RT complication in normal tissues, such as TGF-β and WNT, would be particularly relevant.

## Data Availability Statement

The data presented in the study are deposited in the Gene Expression Omnibus repository, accession number GSE154559.

## Ethics Statement

The studies involving human participants were reviewed and approved by the regional Ethical Committee (CPP Sud-Est, Lyon, France), and cell lines were declared under the numbers DC2008-585 and DC2011-1437 to the Ministry of Research. The patients/participants provided their written informed consent to participate in this study.

## Author Contributions

JL, JD, and MTM designed the study. JD, CK, YM, JR, SM, TVS, and FPC acquired, analyzed, and interpreted the data. JD, JR, FPC, MTM, and JL critically revised the manuscript for important intellectual content. JL and MTM obtained funding. CK, SM, MTM, and JL provided the administrative, technical, and material support. JL is the guarantor of this work and, as such, has full access to all of the data and the accuracy of the data analysis. All authors contributed to the article and approved the submitted version.

## Conflict of Interest

The authors declare that the research was conducted in the absence of any commercial or financial relationships that could be construed as a potential conflict of interest.
