# Supplementary material for: NFATC2 Modulates Radiation Sensitivity in Dermal Fibroblasts From Patients With Severe Side Effects of Radiotherapy
Source: Front Oncol. 2020 Dec 16;10:589168. doi: 10.3389/fonc.2020.589168 (PMC7772431; doi:10.3389/fonc.2020.589168)
Supplement: Supplementary file 1 [file DataSheet_1.docx]

**Legends to supplementary Figures and Tables**

**Figure S1: Gene ontology analysis of differentially expressed genes between patient and control fibroblasts.** Barplot of the 40 most enriched GO biological processes identified with the 445 differentially expressed protein coding genes common between grade 2 vs controls and grade 3 vs controls.

**Figure S2: Hypermethylation of CpG00498368 in fibroblasts from patients who suffered severe radiotherapy side-effects.** (A) Methylation rate of CpG00498368 from global methylome data (N = 8 controls and 16 patients). (B) CpG00498368 methylation state investigated by HRM PCR in 2 controls’ cell strains and 4 patients’ cell strains.

**Figure S3: Effect of 4 different shRNA targeting *NFATC2* on its expression.** *NFATC2* mRNA levels were measured by RTqPCR after infection of control fibroblasts with a lentiviral vector carrying either a shRNA scramble sequence or a shRNA targeting *NFATC2*. sh-*NFATC2*_D were used for next experiments.

**Table S1: List of differentially expressed genes in common between grade 2 patients vs controls and grade 3 patients vs controls.**

**Table S2: List of enriched GO biological process in the 445 differentially expressed protein coding genes in common between grade 2 patients vs controls and grade 3 patients**

**vs controls.**
