# Supplementary figures and images for: NFATC2 Modulates Radiation Sensitivity in Dermal Fibroblasts From Patients With Severe Side Effects of Radiotherapy
Source: Front Oncol. 2020 Dec 16;10:589168. doi: 10.3389/fonc.2020.589168 (PMC7772431; doi:10.3389/fonc.2020.589168)

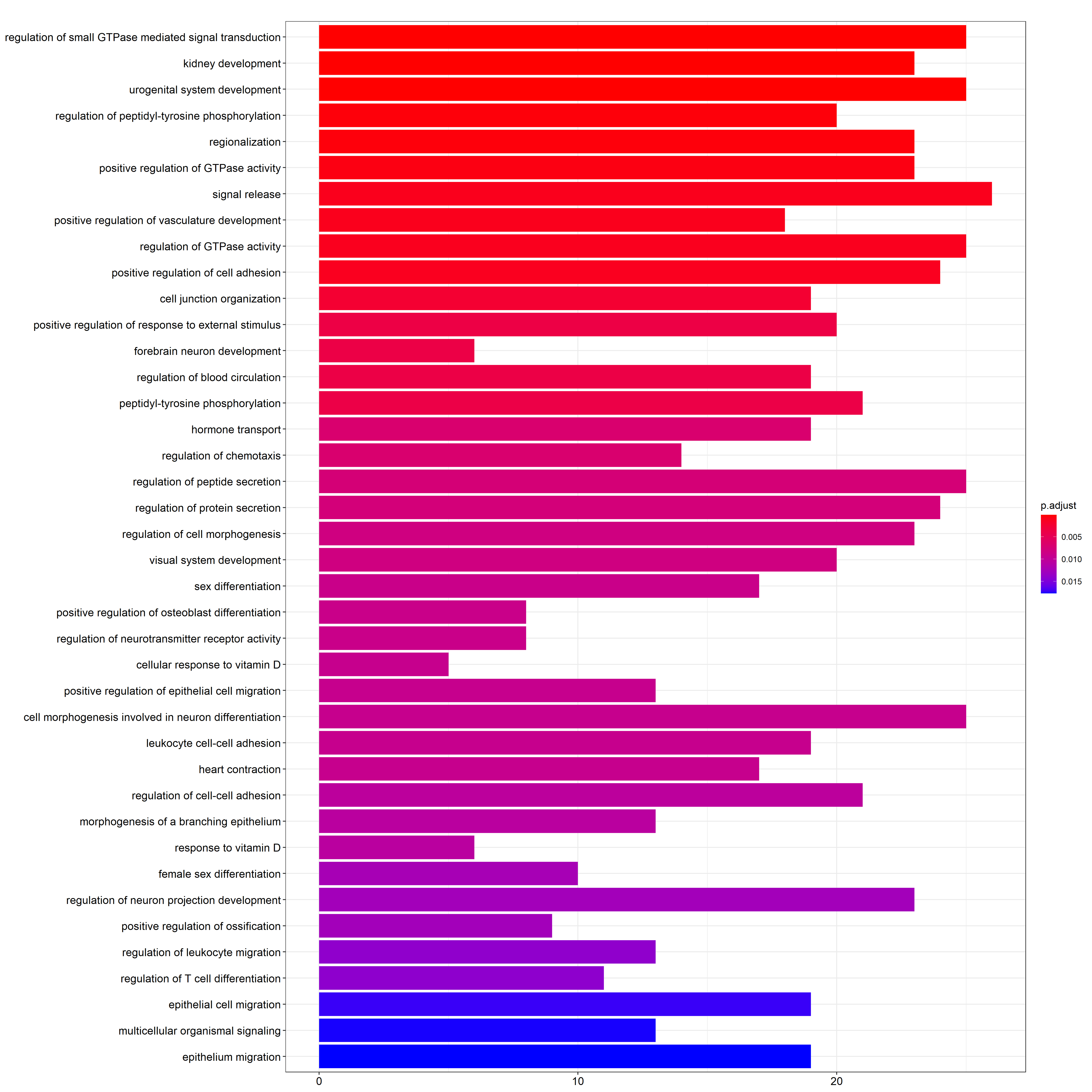

Supplement: Supplementary file 2 [file Image_1.tif]

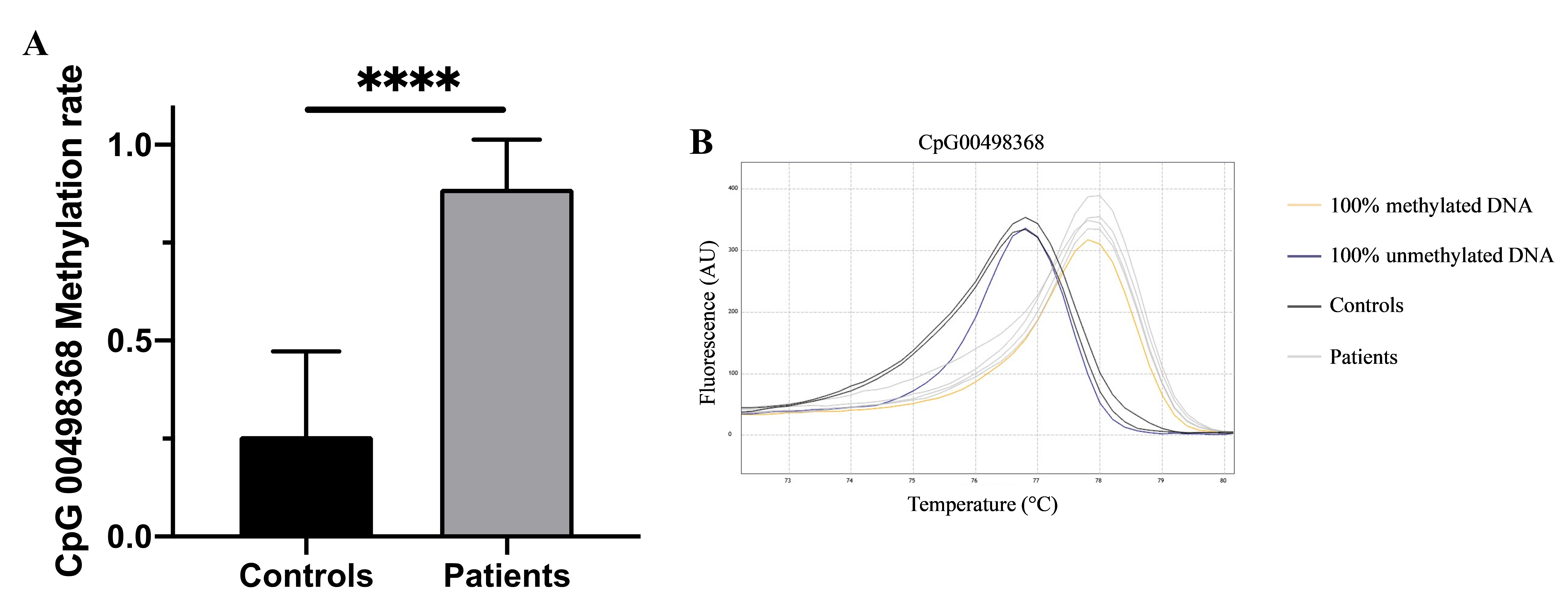

Supplement: Supplementary file 3 [file Image_2.tif]

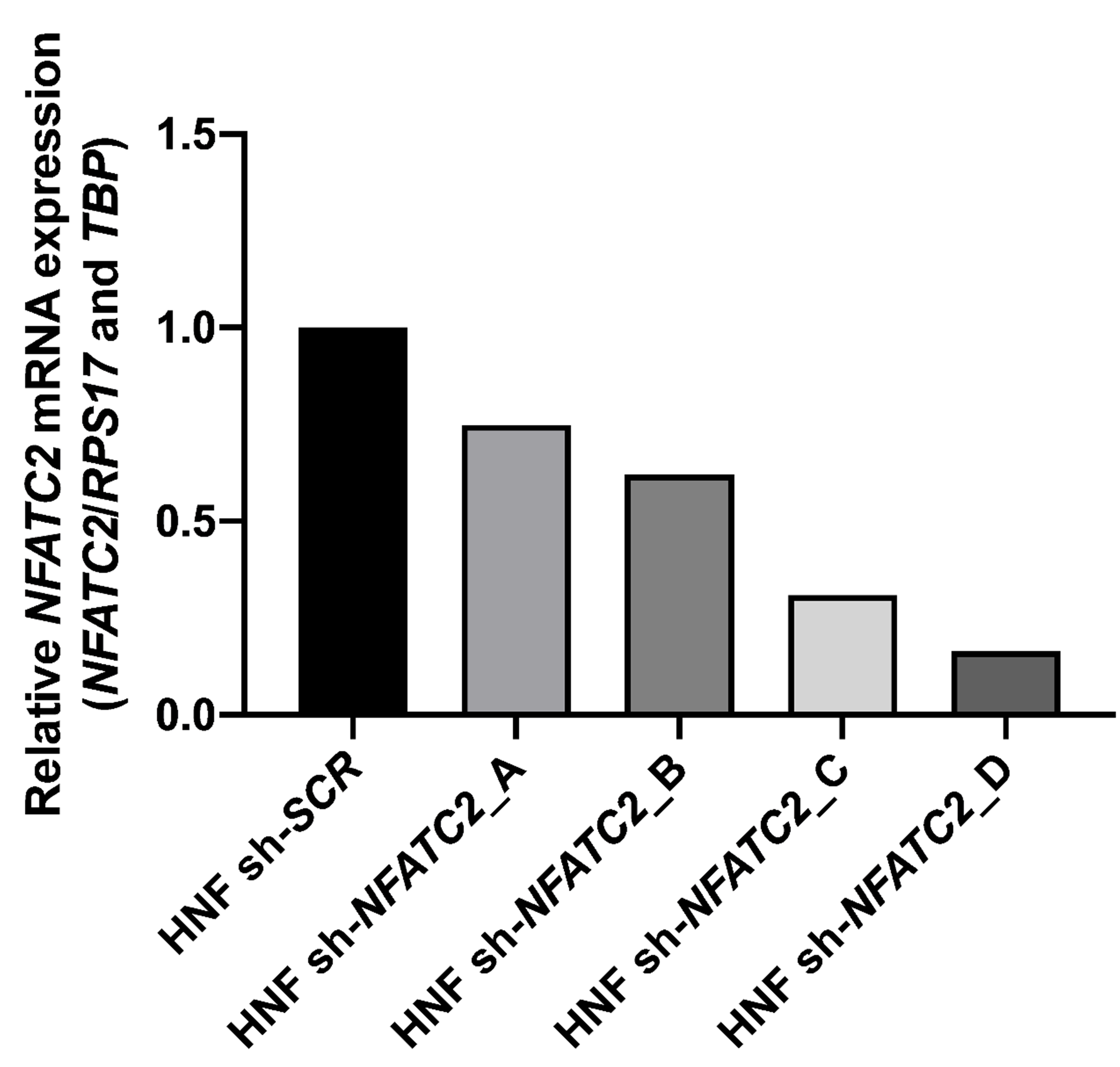

Supplement: Supplementary file 4 [file Image_3.tif]
